# Methicillin-Resistant *Staphylococcus aureus* Sequence Type 239-III, Ohio, USA, 2007–2009^1^

**DOI:** 10.3201/eid1810.120468

**Published:** 2012-10

**Authors:** Shu-Hua Wang, Yosef Khan, Lisa Hines, José R. Mediavilla, Liangfen Zhang, Liang Chen, Armando Hoet, Tammy Bannerman, Preeti Pancholi, D. Ashley Robinson, Barry N. Kreiswirth, Kurt B. Stevenson, for the Prevention Epicenter Program of the Centers for Disease Control and Prevention

**Affiliations:** The Ohio State University Wexner Medical Center, Columbus, Ohio, USA (S.H. Wang, Y. Khan, L. Hines, A. Hoet, P. Pancholi, K.B. Stevenson);; University of Medicine and Dentistry of New Jersey, Newark, New Jersey, USA (J.R. Mediavilla, L. Chen, B.N. Kreiswirth);; University of Mississippi Medical Center, Jackson, Mississippi, USA (L. Zhang, D.A. Robinson);; and The Ohio Department of Health Laboratories, Reynoldsburg, Ohio, USA (T. Bannerman)

**Keywords:** Staphylococcus aureus, methicillin-resistant Staphylococcus aureus, MRSA, MRSA ST239-III, bacteria, sequence type, virulent clones, Brazilian clone, Portuguese clone, Ohio, United States

## Abstract

Identification of virulent strains emphasizes the need for molecular surveillance.

## Medscape CME Activity

Medscape, LLC is pleased to provide online continuing medical education (CME) for this journal article, allowing clinicians the opportunity to earn CME credit. 

This activity has been planned and implemented in accordance with the Essential Areas and policies of the Accreditation Council for Continuing Medical Education through the joint sponsorship of Medscape, LLC and Emerging Infectious Diseases. Medscape, LLC is accredited by the ACCME to provide continuing medical education for physicians.

Medscape, LLC designates this Journal-based CME activity for a maximum of 1 *AMA PRA Category 1 Credit(s)*^TM^. Physicians should claim only the credit commensurate with the extent of their participation in the activity.

All other clinicians completing this activity will be issued a certificate of participation. To participate in this journal CME activity: (1) review the learning objectives and author disclosures; (2) study the education content; (3) take the post-test with a 70% minimum passing score and complete the evaluation at www.medscape.org/journal/eid; (4) view/print certificate.

**Release date: September 20, 2012; Expiration date: September 20, 2013**

## Learning Objectives

Upon completion of this activity, participants will be able to:

- Distinguish the most common strains of methicillin-*resistant Staphylococcus aureus* (MRSA) in the United States
- Assess the clinical characteristics of infection with MRSA-ST239-III
- Analyze the treatment and prognosis of MRSA-ST239-III infection
- Evaluate molecular characteristics of MRSA-ST239-III.

## CME Editor

**Thomas J. Gryczan, MS,** Technical Writer/Editor, *Emerging Infectious Diseases. Disclosure: Thomas J. Gryczan, MS, has disclosed no relevant financial relationships.*

## CME Author

**Charles P. Vega, MD, **Health Sciences Clinical Professor; Residency Director, Department of Family Medicine, University of California, Irvine. *Disclosure: Charles P. Vega, MD, has disclosed no relevant financial relationships.*

## Authors

Disclosures: **Shu-Hua Wang, MD, MPH; Yosef Khan, MBBS, PhD; Jose R. Mediavilla, MBS, MPH; Liangfen Zhang, MD, PhD; Liang Chen, PhD; Armando Hoet, PhD; Tammy Bannerman; D. Ashley Robinson, PhD; Barry N. Kreiswirth, PhD;** and **Kurt B. Stevenson, MD, MPH,** have disclosed no relevant financial relationships. **Lisa Hines, RN, CIC,** *has disclosed the following relevant financial relationships: owns stock, stock options, or bonds from Kimberly-Clark Corp., General Electric Co., Medtronic Inc., Stryker Corp., TEVA Pharmaceutical Industries. ***Preeti Pancholi, PhD*,**** has disclosed the following relevant financial relationships: served as an advisor or consultant for Abbott; served as a speaker or a member of a speakers bureau for Abbott, Nanosphere; received grants for clinical research from Cepheid, Abbott, Quidel, Qiagen, Nanosphere.*
