# Supplementary material for: Methicillin-Resistant Staphylococcus aureus Sequence Type 239-III, Ohio, USA, 2007–2009
Source: Emerg Infect Dis. 2012 Oct;18(10):1557–65. doi: 10.3201/eid1810.120468 (PMC3471631; doi:10.3201/eid1810.120468)
Supplement: Related texts

# Research article: Methicillin-Resistant *Staphylococcus aureus* Sequence Type 239-III, Ohio, USA, 2007–2009^1^

**DOI:** 10.3201/eid1810.120468

**Authors:** Shu-Hua Wang, Yosef Khan, Lisa Hines, José R. Mediavilla, Liangfen Zhang, Liang Chen, Armando Hoet, Tammy Bannerman, Preeti Pancholi, D. Ashley Robinson, Barry N. Kreiswirth, Kurt B. Stevenson, for the Prevention Epicenter Program of the Centers for Disease Control and Prevention

**Affiliations:** The Ohio State University Wexner Medical Center, Columbus, Ohio, USA (S.H. Wang, Y. Khan, L. Hines, A. Hoet, P. Pancholi, K.B. Stevenson);; University of Medicine and Dentistry of New Jersey, Newark, New Jersey, USA (J.R. Mediavilla, L. Chen, B.N. Kreiswirth);; University of Mississippi Medical Center, Jackson, Mississippi, USA (L. Zhang, D.A. Robinson);; and The Ohio Department of Health Laboratories, Reynoldsburg, Ohio, USA (T. Bannerman)

## Abstract

Methicillin-resistant *Staphylococcus aureus* (MRSA) is a human pathogen that has diverse molecular heterogeneity. Most MRSA strains in the United States are pulsed-field gel electrophoresis USA100 sequence type (ST) 5 and USA300 ST8. Infections with MRSA ST239-III are common and found during health care–associated outbreaks. However, this strain has been rarely reported in the United States. As part of a study supported by the Prevention Epicenter Program of the Centers for Disease Control and Prevention (Atlanta, GA, USA), which evaluated transmission of MRSA among hospitals in Ohio, molecular typing identified 78 (6%) of 1,286 patients with MRSA ST239-III infections. Ninety-five percent (74/78) of these infections were health care associated, and 65% (51/78) of patients had histories of invasive device use. The crude case-fatality rate was 22% (17/78). Identification of these strains, which belong to a virulent clonal group, emphasizes the need for molecular surveillance.

*Staphylococcus aureus* is a major human pathogen that possesses multiple toxins and virulence mechanisms (*1*). Antimicrobial drug resistance in *S. aureus* has added to the complexity of treating serious infections caused by this bacteria, and methicillin-resistant *S. aureus* (MRSA) appears to have greater virulence than methicillin-susceptible strains (*2*,*3*). Most MRSA strains in the United States are pulsed-field gel electrophoresis (PFGE) types USA100 and USA300, corresponding to multilocus sequence typing (MLST) ST5 and ST8, respectively (*4*). MRSA belonging to MLST ST239 and harboring staphylococcal cassette chromosome *mec* (SCC*mec*) type III (MRSA ST239-III) are associated with infections in health care settings, outbreaks, increased resistance to antimicrobial drugs, and capacity for invasive disease (*5*–*7*).

MRSA ST239-III has a history of successful dissemination in many regions, leading to a diverse array of regionally prevalent clones. These clones include the Brazilian; British Epidemic 1, 4, 7, 9, and 11; Canadian Epidemic 3/Punjab; Czech; Eastern Australian 2 and 3; Georgian; Hungarian; Lublin; Nanjing/Taipei (ST241); Portuguese; and Vienna clones (*8*,*9*). Although it is common worldwide, MRSA ST239-III has not played any predominant role in the United States; infections with MRSA ST239-III have been rarely reported in the United States since the 1990s (*9*–*13*). Recently, only 2 reports of this strain in the United States involving sporadic nasal colonization and bloodstream infections have been published (*13*,*14*).

In this study, we describe clinical epidemiologic characteristics and molecular analysis of clinical infections with MRSA ST239-III in the midwestern United States. Identification of a strain from such a virulent clonal group in the United States with wide dissemination in other parts of the world represents a potential public health concern.

## Methods

### Sampling Method

As part of Centers for Disease Control and Prevention (CDC) (Atlanta, GA, USA)–sponsored Prevention Epicenter Program study evaluating the transmission of MRSA between hospitals in Ohio, USA, molecular analysis was performed on a group of clinical MRSA isolates collected from The Ohio State Health Network (OSHN). The OSHN consists of The Ohio State University (OSU) Wexner Medical Center (WMC), which is a tertiary care medical center, and 7 smaller community hospitals located 30–120 miles from OSU. All MRSA specimens from community hospitals and selected OSU MRSA blood isolates and isolates from patients residing in the catchment areas of the outreach hospitals were prospectively collected during March 2009–February 2010 for genotyping by using a repetitive element PCR (rep-PCR). Among archived MRSA isolates from OSUWMC from January 2007 through February 2009, only a selection of isolates was chosen for genotyping (not a randomized sampling). The total number of isolates and the total number of ST239 from each time period was collected.

### Data Collection

We performed medical record reviews for 1,286 patients. Patient demographic characteristics (presence of health care–related risk factors during the preceding 12 months, presence of an invasive device during the previous 7 days, and concurrent conditions) were collected. Patient-level data, including addresses for geocoding, were entered into a secure database within the OSUWMC Information Warehouse.

### Classification of MRSA Infections

All MRSA cases were classified into 3 categories on the basis of accepted epidemiologic definitions (*11*). The first category was health care–associated, defined as a culture obtained >48 hours after admission. The second category was health care–associated community onset, defined as a culture obtained <48 hours after admission with identified health care–associated risk factors. The third category was community-associated, defined as a culture obtained <48 hours after admission without health care–associated risk factors. Health care–associated risk factors comprised presence of an invasive device, history of MRSA infection or colonization, surgery, hospitalization, dialysis, or residence in a long-term care facility in the 12 months preceding the culture.

Outcomes for MRSA infection were categorized as cure (complete resolution after antimicrobial drug treatment); failure (persistence of infection and change in antimicrobial drug regimen); relapse (resolution of infection after complete treatment with subsequent development of new symptoms); recurrent (redevelopment of MRSA at same or other site >2 weeks after completion of treatment for initial MRSA infection); indeterminate (unknown outcome); and death (death <30 days after diagnosis of MRSA infection because of any cause or during the same hospitalization). Destination after hospital discharge, such as home or skilled nursing facility, was also noted.

### Drug Susceptibility Testing

The respective OSHN Clinical Microbiology Laboratories initially identified all MRSA isolates by using standard microbiological methods. Antimicrobial drug susceptibility testing was performed at each institution, and results were interpreted according to Clinical and Laboratory Standards Institute break point guidelines (*15*). At OSUWMC, antimicrobial drug susceptibility testing was performed by using the automated Micro-Scan method (Siemens Diagnostics, Sacramento, CA, USA), and only constitutive clindamycin testing was performed. Linezolid MIC >4 mg/L were confirmed by using the Etest method (bioMérieux, Marcy l’Etoile, France).

### Genotyping

The MRSA ST239 III isolates were genotyped initially by using rep-PCR, followed by PFGE, staphylococcal protein A sequencing (*spa* typing), SCC*mec* typing, and *mec*-associated direct repeat unit (*dru*) typing. Selected isolates were also characterized by MLST and single-nucleotide polymorphism (SNP) typing. Detection of genes encoding Panton–Valentine leukocidin (PVL), toxic shock syndrome toxin (TSST), arginine catabolic mobile element (ACME), and high-level mupirocin resistance (*mupA*) was also performed. Brief descriptions of each testing method are outlined below.

#### rep-PCR

The DiversiLab System (bioMérieux, Durham, NC, USA) was used for rep-PCR analysis according to described methods (*16*). Isolates belonging to designated rep-PCR clusters shared >95% similarity. In addition, comparison of matching patterns in the DiversiLab System library was initially used to infer the PFGE and SCC*mec* types, which were later validated by using appropriate testing methods. The numeric classification system used for rep-PCR analysis is unique to OSUWMC.

#### PFGE

The PulseNet protocol for molecular subtyping of *S*. *aureus* was followed. *Salmonella enterica* serotype Braenderup DNA was digested with *Xba*I (Roche, Indianapolis, IN, USA) and used as the normalization standard for gel analysis. *S. aureus* chromosomal DNA was digested with *Sma*I (Roche). Fragments were separated in a clamped homogenous electric field mapper unit (Bio-Rad Laboratories, Hercules, CA, USA). Fingerprint images were analyzed by using Bionumerics software version 4.61 (Applied Maths NV, Sint-Martens-Latem, Belgium). The traditional classification of PFGE subtypes was not used because we were not analyzing for an outbreak (*17*). Thus, interpretations of possibly or probably related PFGE subtypes between strains obtained by PFGE band patterns were not made. Each PFGE band difference was classified as a unique PFGE pattern.

#### *spa* Typing

*spa* typing (*18*) was performed on all MRSA isolates by using eGenomics software (www.egenomics.com) as described (*19*); Ridom *spa* types were subsequently assigned by using the SpaServer website (www.spaserver.ridom.de).

#### SCC*mec* Typing

SCC*mec* typing was performed on all MRSA isolates by using a described multiplex real-time PCR (*20*). This PCR is specific for 2 essential gene complexes (*ccr* and *mec*) found in all SCC*mec* elements.

#### *dru* Typing

MRSA isolates were also characterized by sequencing the hypervariable *dru* repeat region within the SCC*mec* element (*21*) and using DruID software (*9*). New *dru* types were submitted to www.dru-typing.org.

#### MLST and PVL, TSST, ACME, and Mupirocin Resistance

MLST was performed on representative isolates as described (*22*) by using the MLST database (http://saureus.mlst.net). PCR-based detection of PVL (*23*), TSST (*24*), ACME (*25*), and *mupA* (*26*) was performed on all isolates as described.

#### SNP Typing

A panel of 43 SNPs for describing the global population structure of MRSA ST239-III (*9*) was used to identify the haplotypes of 22 isolates. These SNPs were typed by using Golden-Gate Genotyping Assay (Illumina, San Diego, CA, USA) and conventional Sanger sequencing.

### Statistical Analysis

All patient demographic, clinical, and molecular typing data were aggregated in tabular format, and descriptive statistics were generated by using SAS version 9.2 (SAS Institute Inc., Cary NC, USA), for demographic and risk factor history. A significant difference between ST239 and other strains (US300, US100, and all other strains) was examined by using χ^2^ tests for categorical variables and *t*-tests for continuous variables. An α level of 0.5 was used.

### Human Subjects Protection

We obtained approval for this study from the OSU Office of Responsible Research Practices’ Biomedical Institutional Review Board. The OSU Information Warehouse has established honest broker status with the OSU Institutional Review Board, enabling storage of fully identifiable data and presentation of patient data to investigators in a coded format that maintains patient confidentiality.

## Results

### Patient Characteristics

Of 1,286 clinical MRSA isolates, 78 (6%) were identified as MRSA ST239-III; 71 (91%) were obtained from OSU and 7 (9%) from community hospitals. Seven (2%) of 397 isolates were obtained from outreach community hospitals, 37 (6%) of 613 from OSUWMC, and 34 (12%) of 276 from OSUWMC archives.

These strains were first recognized by rep-PCR as possible SCC*mecA* type III isolates from the DiversiLab System library with imputed Brazilian PFGE types. Additional molecular typing identified MRSA ST239-III in a clade different from that containing the Brazilian strains.

Demographics and clinical characteristics of 78 patients infected with MRSA ST239-III are shown in the Table. Among patients with MRSA ST239-III infections, 46 (59%) were male, 65 (83%) were white, 31 (40%) disabled, and 29 (37%) were retired. Seventy-four (95%) had health care–associated isolates and 51 (65%) had histories of use of invasive devices within the previous 7 days. Distribution of specimen types include 37 (47%) bloodstream infections (BSIs), 19 (24%) lower respiratory tract infections (LRTIs), 11 (14%) skin and soft tissue infections, and 11 (14%) other infections. Seventeen (22%) patients died, and 18 (23%) patients had treatment failures, recurring infections, or relapses. Only 32% of the patients were discharged home.

Comparison of clinical characteristics of MRSA ST239-III with those of PFGE types USA100 and USA300 and other non–ST239 infections are shown in the Table. Most ST239 isolates were health care–associated MRSA and had characteristics and concurrent conditions similar to those associated with USA100. MRSA ST239-III did not have virulent determinants often associated with health care–associated or community-associated MRSA strains, such as PVL, TSST, ACME, or *mupA*.

### Characteristics of MRSA ST239-III Isolates

Resistance was observed to clindamycin (76/76, 100%), moxifloxacin (47/47, 100%), gentamicin (74/77, 96%), tetracycline (70/74, 95%), and trimethoprim–sulfamethoxazole (62/77, 81%). All MRSA ST239-III isolates were susceptible to vancomycin. For daptomycin, 64 (98%) of 65 isolates were susceptible, and 1 blood isolate with an MIC of 2 mg/L was classified as uninterpretable. For linezolid, 69 (97%) of 71 isolates were susceptible and 2 blood isolates with MICs >4 mg/L were classified as uninterpretable.

### Molecular Typing of MRSA ST239-III Isolates

Eight rep-PCR patterns (6, 19, 22, 42, 53, 78, 79, and 97) were detected among MRSA ST239-III isolates, and 39 (50%) isolates had rep-PCR pattern 6. Similarly, isolates were distributed among 9 PFGE patterns (A, B, C, D, E, F, G, H, and I), and 58 (74%) had PFGE pattern A. The rep-PCR and PFGE patterns are shown in Figure 1. PFGE pattern F is <80% similar with other PFGE subtypes in the dendrogram and was identified as an unknown PFGE type by the CDC database. However, molecular testing confirmed it to be MRSA ST239-III: MLST 239, SCC*mec* type III, *spa* type 3(t037), *dru* type dt15b, and SNP haplotype H9.

**Figure 1:** Methicillin-resistant *Staphylococcus aureus* sequence type 239-III isolates, Ohio, USA, 2007–2009, based on A) repetitive element PCR (rep-PCR) and B) pulsed-field gel electrophoresis. Virtual gel results are shown for 8 DiversiLab System (bioMérieux, Durham, NC, USA) rep-PCR patterns. Pattern numbers assigned are unique to the Ohio State University Wexner Medical Center.

Four *spa t*ypes, including eGenomics *spa* types 3 (Ridom t037), 314 (t363), 121 (t421), and 1256 (t631) were identified, and 74 (95%) isolates were classified as *spa* type 3 (t037). In contrast, isolates were grouped into 13 *dru* types (dt2c, dt6n, dt9g, dt12i, dt13k, dt14 g, dt15b, dt15h, dt15i, dt15j, dt15k, dt16a, and dt23a), and 60 (77%) isolates were classified as dt15b. MLST typing of selected representative isolates confirmed 13 to be MRSA ST239-III (2-3-1-1-4-4-3), 3 as ST1801 (2-3-1-1-4-19-3), and 1 as ST2017 (2-3-1-1-4-19-227). ST1801 is a single-locus variant of MRSA ST239-III, and ST2017 is a novel single-locus variant of ST1801 and a double-locus variant of MRSA ST239. All were SCC*mec* type III, and the *ccrC* locus was not detected. The genes that encode PVL, TSST, ACME, or high-level mupirocin resistance were not found in any isolates.

A total of 33 unique genotypic combinations from 78 patients infected with MRSA ST239-III were represented among isolates with complete SCC*mec*, rep-PCR, PFGE, *spa*, and *dru* data. This information and year of isolation are shown in Figure 2. The genotype cluster sizes ranged from 2 to 18 isolates; 23 of the isolates were unique. In contrast, SNP typing of 22/78 isolates with a panel of 43 SNPs showed them to be indistinguishable from each other. All isolates tested belonged to haplotype 9 (H9) within MRSA ST239-III clade II (Figure 3). This clade was composed of isolates from many continents, including many from sources in Asia (*9*).

**Figure 2:** Molecular characteristics of 78 methicillin-resistant *Staphylococcus aureus* sequence type 239-III isolates, Ohio, USA, 2007–2009. SCC*mec*, staphylococcal cassette chromosome *mec*; *spa*, staphylococcal protein A; rep-PCR, repetitive element PCR; PFGE, pulsed-field gel electrophoresis; *dru*, direct repeat unit; dt, *dru* type. The 33 unique genotypic combinations are contrasted by black and gray shading.

**Figure 3:** Single-nucleotide polymorphism (SNP) haplotype map showing position of methicillin-resistant *Staphylococcus aureus* sequence type 239-III (MRSA ST239-III) isolates, Ohio, USA, 2007–2009, within the global population structure of the MRSA ST239-III clonal group. Circles indicate distinct haplotypes, as defined by using a panel of 43 SNPs (*9*). Sizes of circles indicate relative frequency of different haplotypes. Arrows indicate haplotype 5 (H5), which includes the Brazilian clone, and haplotype 9 (H9), which includes the 22 MRSA ST239-III isolates from Ohio. Relationships between haplotypes were determined by using maximum-parsimony analysis (*9*).

## Discussion

MRSA ST239-III has demonstrated epidemic potential worldwide, and identification of this strain in the United States might represent a major public health concern. Although the precise time frame of emergence of this strain in Ohio is unknown, it appears to have been present before the study because it was identified in archived specimens dating back to 2007. The origin of the MRSA ST239-III clonal group has been dated to the mid-20th century in 2 studies (*9*,*27*). Thus, the Ohio strain might have been introduced into the region anytime during the past 40 years. Because of incomplete sampling during the study, the transmission dynamics of Ohio MRSA ST239 III are uncertain. Whether the prevalence would have been >6% if complete sampling was conducted is unknown. Furthermore, we found no epidemiologic evidence of clustering of cases consistent with an outbreak of infection with this strain. The large number of isolates at OSUWMC merely reflects its role as a large referral center.

Clinically, MRSA ST239-III is primarily health care associated, causes major outbreaks, shows increased resistance to antimicrobial drugs, and demonstrates a capacity for causing invasive disease (*5*–*7*,*28*,*29*). Comparison of characteristics of patients infected with MRSA ST239-III with those infected with PFGE USA300, PFGE USA100, and other strains showed that MRSA ST239-III is similar to USA100 in terms of health care–associated risk factors and outcomes (Table).

**Table:** Characteristics of 1,286 patients with MRSA ST239-III and non–MRSA ST239-III infections, Ohio, USA, 2007–2009*

Characteristic	ST239, n = 78	USA100, n = 481	p value	USA300, n = 574	p value	All other, n = 153	p value
Outreach hospital isolates	7 (9)	81 (17)	NA	262 (46)	NA	47 (31)	NA
Patient demographics							
Mean age, y (range)	58 (19–90)	61 (18–99)	0.06	43 (18–92)	<0.0001	49 (18–94)	0.001
Male sex	46 (59)	259 (54)	0.39	311 (54)	0.42	80 (52)	0.33
White race	65 (83)	393 (82)	0.85	441 (77)	0.61	126 (82)	0.77
Medical history							
Diabetes	33 (42)	149 (31)	0.047	116 (20)	<0.0001	35 (23)	0.002
Chronic lung disease	26 (33)	117 (24)	0.09	74 (13)	<0.0001	17 (11)	<0.0001
Renal failure	19 (24)	93 (19)	0.39	37 (6)	<0.0001	17 (11)	0.008
Malignancy	13 (17)	97 (20)	0.47	48 (8)	0.02	30 (20)	0.58
Health care–associated risk factors, past 12 mo							
Hospitalization	58 (74)	296 (62)	0.03	148 (26)	<0.0001	58 (38)	<0.0001
Use of invasive device	35 (45)	168 (35)	0.09	66 (12)	<0.0001	35 (23)	0006
Surgery	35 (45)	179 (37)	0.19	79 (14)	<0.0001	33 (22)	0.0002
History of MRSA infection	26 (33)	77 (16)	0.0003	82 (14)	<0.0001	29 (19)	0.015
Stay in long-term care facility	22 (28)	155 (32)	0.47	34 (6)	<0.0001	14 (9)	0.0002
Hemodialysis	15 (19)	47 (10)	0.068	18 (3)	<0.0001	10(7)	0.015
Other	10 (13)	29 (6)	0.03	40 (7)	0.06	7 (5)	0.02
Invasive devices <7 d before infection							
Central venous catheter	25 (32)	156 (32)	0.94	61 (11)	<0.0001	32 (21)	0.06
Foley catheter	17 (22)	99 (21)	0.8	55 (10)	0.001	17 (11)	0.03
Hemodialysis	13 (17)	49 (10)	0.02	19 (3)	<0.0001	8 (5)	0.008
Mechanical ventilation	14 (18)	85 (18)	0.95	35 (6)	0.0002	11 (7)	0.012
Drainage tubes	8 (10)	50 (10)	0.97	10 (2)	<0.0001	5 (3)	0.02
Total parenteral nutrition	8 (10)	22 (5)	0.04	4 (1)	<0.0001	0	0.0001
Other	20 (26)	71 (15)	0.016	39 (7)	<0.0001	15 (10)	0.0015
Classification of MRSA infection							
Health care associated	32 (41)	197 (41)	0.97	63 (11)	<0.0001	37 (24)	0.01
Health care associated community onset	42 (54)	214 (44)	0.12	113 (20)	<0.0001	49 (32)	0.0012
Community associated	4 (5)	70 (15)	0.02	398 (69)	<0.0001	67 (44)	<0.0001
Outcome							
Cure	23 (29)	149 (31)	0.81	94 (16)	0.005	38 (25)	0.49
Failure	3 (4)	7 (1.5)	0.14	12 (2)	0.33	5 (3)	0.81
Relapse	4 (5)	7 (1.5)	0.03	2 (1)	0.0001	1 (1)	0.022
Recurrent	11 (14)	45 (9)	0.2	36 (6)	0.012	3 (2)	0.0002
Indeterminate	20 (26)	192 (40)	0.015	408 (71)	0.001	97 (63)	0.0001
Death	17 (22)	81 (17)	0.29	22 (4)	0.001	9 (6)	0.0003
No. patients admitted	74	429	0.005	310	0.0003	111	0.003
Admitting service							
Intensive care unit	14 (19)	58 (14)	0.2	29 (9)	0.019	9 (8)	0.047
Medicine service	31 (42)	238 (55)	0.03	203 (66)	0.0002	75 (68)	0.0007
Surgery service	20 (27)	117 (27)	0.95	59 (19)	0.12	20 (18)	0.126
Other specialty care unit†	9 (12)	16 (4)	0.002	19 (6)	0.073	7 (6)	0.16
Destination after discharge							
Home	20 (27)	146 (34)	0.24	209 (67)	<0.0001	60 (54)	0.0003
Another facility, long-term care center, or rehabilitation center	35 (47)	204 (48)	0.97	79 (24)	<0.0001	40 (36)	0.12
Median duration of hospitalization,‡ d (range)	16 (1–143)	11 (0–136)	0.07	6 (0–169)	<0.0001	9 (1–124)	0.01

*Values are no. (%) unless otherwise indicated. MRSA, methicillin-resistant *Staphylococcus aureus*; ST, sequence type; NA, not applicable. The p-values were generated to assess whether differences in demographic and risk factor history existed between ST239 and other strains (USA100, USA300, all other strains). χ^2^ tests were used for categorical variables and *t*-tests were used for continuous variables. †Other specialty care unit admitting services included bone marrow, cardiology, hematology, surgical oncology, transplant, and obstetrics and gynecology. ‡Duration of hospitalization and days to MRSA culture–positive included only inpatients; outpatient visits such as clinic or emergency department visits were excluded from the analysis. Duration of hospitalization was calculated from the date of the current hospital admission to the date of discharge. Hospitalization days at another institution before transfer were not included.

Concern for the invasive potential of MRSA ST239-III was illustrated during a 2-year intensive care unit (ICU) outbreak in London in which patients infected with MRSA ST239-III were more likely than patients with non–ST239 strains to show development of bacteremia (47% vs 13%; p<0.001) and have culture-positive vascular access device infections (59% vs 26%; p<0.001) (*29*). In our study, 19% (14/74) of patients infected with MRSA ST239-III strains were admitted to ICU, compared with 11% (96/850) of patients infected with non–ST239 strains (Table). Among MRSA ST239-III ICU admissions, 50% (7/14) were BSIs and 50% (7/14) were LRTIs. The crude mortality rate for persons with BSIs and LRTIs was 22% (8/37) and 47% (9/19) respectively. In our study population, MRSA ST239-III had a higher case-fatality rate than did USA300 (4%) and USA100 (17%) (Table).

Increased illness and death for pulmonary disease might be caused by an enhanced ability of MRSA ST239-III strains to produce biofilms and adhere to airway epithelial cells, providing a biologically plausible explanation for its potential to cause pneumonia and central line–associated BSIs (*28*,*29*). The association of MRSA ST239-III isolates with pulmonary infections has also been reported in South Korea (*30*). Moreover, in a multicenter study involving 21 hospitals in Beijing, where MRSA ST239-III is especially prevalent, 61% of MRSA LRTI cases were attributed to MRSA ST239-III (*31*). The London outbreak strain of MRSA ST239-III, and most examined MRSA ST239-III strains from the People’s Republic of China, belong to the same clade (clade II) as the Ohio strain of MRSA ST239-III, demonstrating the potential for this lineage to disseminate and cause disease (*9*,*27*,*32*). Because of incomplete collection of all MRSA strains causing LRTIs, whether MRSA ST239-III is the prevalent strain in MRSA pneumonias is unknown. A total of 17% (219/1,286) of the study isolates were pulmonary infections and 9% (19/219) of the pneumonias were caused by MRSA ST239-III.

PFGE is the standard for MRSA outbreak investigations. However, we chose a rapid PCR–based method that could serve as a potential screening surveillance tool to identify outbreaks of a particular strain. Although many institutions might be using PFGE for outbreak investigations to identify clonal clusters, methods for PFGE subtype classification are often not standardized between laboratories (*33*,*34*). To maximize uniformity and enable comparisons of PFGE classifications between institutions, the standardized CDC protocols for performing and analyzing PFGE results were used. The initial CDC PFGE analysis classified our isolates as the Brazilian clone. However, SNP typing unambiguously placed the Ohio isolates within a different clade than the Brazilian clone (Figure 3) (*9*). Because PFGE typing cannot define specific lineages for MRSA ST239-III, CDC has changed the reporting of MRSA ST239-III strains from Brazilian subtype to ST239. Laboratories will need to perform additional testing to identify specific clades or clonal groups. Classification of the 78 MRSA ST239-III isolates by the traditional PFGE outbreak method would identify only 2 PFGE subtypes instead of the 8 observed patterns. PFGE subtype 1 would consist of isolates A–E, G, and H, and subtype 2 would consist of isolate F (Figure 1, panel B).

Classification issues also exist with respect to the DiversiLab system. Five of the 8 initial rep-PCR patterns matched isolates in the DiversiLab library, which likewise categorized them as the Brazilian clone. In contrast, 3 other rep-PCR patterns did not match any isolates in the DiversiLab library. Additional molecular tests were performed to identify these isolates as MRSA ST239-III.

Because of the novel identification of MRSA ST239-III strains, we performed a battery of molecular tests to better classify the strains. Although the same *dru* types might be found in unrelated MRSA lineages and different staphylococcal species (*32*), variation at the *dru* locus within the SCC*mec* III element is consistent with MRSA ST239-III phylogeny (*9*). Because use of this typing method is relatively new, further study of its phylogenetic utility is needed (*21*). The feasibility of population-based genome-wide SNP datasets has been demonstrated for MRSA ST239-III by Harris et al. (*27*). Cluster groupings from these PFGE, rep-PCR, and *dru* methods were not identical, suggesting that the molecular tests are not completely interchangeable. Of the various molecular methods used to index genomic variation in MRSA, *dru* typing and rep-PCR appeared to be more discriminatory.

The publication of only 2 recent reports of MRSA ST239-III in the United States (*13*,*14*) might have resulted from inadequate national surveillance or low transmissibility of the strain. Institutions might not be able to perform genotyping on all their isolates because of lack of appropriate laboratory facilities or resources. An alternative method of surveillance for MRSA ST239-III might be evaluation of the drug susceptibility pattern. Most of the MRSA ST239-III strains in our study were resistant to clindamycin, tetracycline, and gentamicin. In a subset analysis, the phenotypic patterns of antimicrobial drug susceptibility and MRSA genotype prediction were determined for 798 MRSA isolates from the OSHN dataset. Ninety-four percent (63/67) of the MRSA ST239-III isolates were resistant to all 3 drugs (clindamycin, tetracycline, and gentamicin) (*35*). Use of phenotypic drug susceptibility patterns might alert infection control practitioners that they are dealing with a MRSA ST239-III clone. Additional molecular testing, such as *spa* typing in conjunction with SCC*mec* typing, can then be performed to confirm the strain type.

We have genotyped and geocoded 1,286 MRSA isolates in the CDC Prevention Epicenter Study by using rep-PCR. The lack of a clear association between MRSA ST239-III molecular genotype patterns could be caused by the retrospective nature of this study, lack of complete population sampling, and the inability to gather social network history. The rep-PCR and PFGE methods might be of limited use in evaluating geographic clustering at the scale studied here unless specific lineages of the reference strains are included in the respective databases. Because of the clonality exhibited by MRSA ST239-III, even a relatively small panel of 43 SNPs is able to identify the same major phylogenetic lineages that are identified by genome-wide SNPs. A more detailed genome-wide SNP analysis might be required to resolve geographic clustering. Additional social networking for determining spatial, temporal, and geographic relationships of patients at the medical institutions studied is under way to identify potential nosocomial interhospital and intrahospital transmission.

Recent identification of a strain of MRSA ST239-III in the midwestern United States is a major public health concern. Globally, this strain has demonstrated increased virulence and widespread dissemination. Because of an inadequate national surveillance system, temporal emergence and dissemination of the strain in the United States is uncertain. The MRSA ST239-III strains in Ohio have molecular heterogeneity and geographic diversity. The SNP-based data suggest that the strains also show clonal diversity. Continued surveillance is warranted because this MRSA ST239-III strain, similar to related strains worldwide, exhibits increased antimicrobial drug resistance, capacity for causing invasive disease, potential for causing outbreaks, and resulting in illnesses and deaths. MRSA ST239-III strains might have the potential to become established locally and disseminate among health care institutions as reported in other regions. Our study underscores the value of molecular surveillance, including traditional fingerprinting methods and newer sequence-based typing methods, as a critical component in understanding the evolving epidemiology of MRSA.

## References

[1] Lowy FD. *Staphylococcus aureus* infections. N Engl J Med. 1998;339:520–32. 10.1056/NEJM1998082033908069709046

[2] Cosgrove SE, Sakoulas G, Perencevich EN, Schwaber MJ, Karchmer AW, Carmeli Y. Comparison of mortality associated with methicillin-resistant and methicillin-susceptible *Staphylococcus aureus* bacteremia: a meta-analysis. Clin Infect Dis. 2003;36:53–9. 10.1086/34547612491202

[3] Melzer M, Eykyn SJ, Gransden WR, Chinn S. Is methicillin-resistant *Staphylococcus aureus* more virulent than methicillin-susceptible *S. aureus*? A comparative cohort study of British patients with nosocomial infection and bacteremia. Clin Infect Dis. 2003;37:1453–60. 10.1086/37932114614667

[4] McDougal LK, Steward CD, Killgore GE, Chaitram JM, McAllister SK, Tenover FC. Pulsed-field gel electrophoresis typing of oxacillin-resistant *Staphylococcus aureus* isolates from the United States: establishing a national database. J Clin Microbiol. 2003;41:5113–20. 10.1128/JCM.41.11.5113-5120.200314605147PMC262524

[5] Feil EJ, Nickerson EK, Chantratita N, Wuthiekanum V, Srisomang P, Cousind R, Rapid detection of the pandemic methicillin-resistant *Staphylococcus aureus* clone ST 239, a dominant strain in Asian hospitals. J Clin Microbiol. 2008;46:1520–2. 10.1128/JCM.02238-0718234867PMC2292922

[6] Holden MT, Lindsay JA, Corton C, Quail MA, Cockfield JD, Pathak S, Genome sequence of a recently emerged, highly transmissible, multi-antibiotic- and antiseptic-resistant variant of methicillin-resistant *Staphylococcus aureus*, sequence type 239 (TW). J Bacteriol. 2010;192:888–92. 10.1128/JB.01255-0919948800PMC2812470

[7] Aires de Sousa M, Conceição T, Simas C, de Lencastre H. Comparison of genetic backgrounds of methicillin-resistant and -susceptible *Staphylococcus aureus* isolates from Portuguese hospitals and the community. J Clin Microbiol. 2005;43:5150–7. 10.1128/JCM.43.10.5150-5157.200516207977PMC1248511

[8] Monecke S, Coombs G, Shore AC, Coleman DC, Akpaka P, Borg M, A field guide to pandemic, epidemic and sporadic clones of methicillin-resistant *Staphylococcus aureus.* PLoS ONE. 2011;6:e17936. 10.1371/journal.pone.001793621494333PMC3071808

[9] Smyth DS, McDougal LK, Gran FW, Manoharan A, Enright MC, Song JH, Population structure of a hybrid clonal group of methicillin-resistant *Staphylococcus aureus*, ST239-MRSA-III. PLoS ONE. 2010;5:e8582. 10.1371/journal.pone.000858220062529PMC2797301

[10] de Lencastre H, Severina EP, Roberts RB, Kreiswirth BN, Tomasz A. Testing the efficacy of a molecular surveillance network: methicillin-resistant *Staphylococcus aureus* (MRSA) and vancomycin-resistant *Enterococcus faecium* (VREF) genotypes in six hospitals in the metropolitan New York City area. The BARG Initiative Pilot Study Group. Bacterial Antibiotic Resistance Group. Microb Drug Resist. 1996;2:343–51. 10.1089/mdr.1996.2.3439158794

[11] Klevens RM, Morrison MA, Nadle J, Petit S, Gershman K, Ray S, Invasive methicillin-resistant *Staphylococcus aureus* infections in the United States. JAMA. 2007;298:1763–71. 10.1001/jama.298.15.176317940231

[12] Chung M, Dickinson G, de Lencastre H, Tomasz A. International clones of methicillin-resistant *Staphylococcus aureus* in two hospitals in Miami, Florida. J Clin Microbiol. 2004;42:542–7. 10.1128/JCM.42.2.542-547.200414766814PMC344518

[13] Tenover FC, Tickler IA, Goering RV, Kreiswirth BN, Mediavilla JR, Persing DH. Characterization of nasal and blood culture isolates of methicillin-resistant *Staphylococcus aureus* from patients in United States hospitals. Antimicrob Agents Chemother. 2012;56:1324–30. 10.1128/AAC.05804-1122155818PMC3294931

[14] Mermel LA, Eells SJ, Acharya MK, Cartony JM, Dacus D, Fadem S, Quantitative analysis and molecular fingerprinting of methicillin-resistant *Staphylococcus aureus* nasal colonization in different patient populations: a prospective, multicenter study. Infect Control Hosp Epidemiol. 2010;31:592–7. 10.1086/65277820402589

[15] Clincal and Laboratory Standards Institute. Performance standards for antimicrobial susceptibility testing. 19th informational supplement, vol 29, no. 3. M100–S19. Wayne (PA): The Institute; 2009.

[16] Healy M, Huong J, Bittner T, Lising M, Frye S, Raza S, Microbial DNA typing by automated repetitive-sequence-based PCR. J Clin Microbiol. 2005;43:199–207. 10.1128/JCM.43.1.199-207.200515634972PMC540112

[17] Tenover FC, Arbeit RD, Goering RV, Mickelsen PA, Murray BE, Persing DH, Interpreting chromosomal DNA restriction patterns produced by pulsed-field gel electrophoresis: criteria for bacterial strain typing. J Clin Microbiol. 1995;33:2233–9.749400710.1128/jcm.33.9.2233-2239.1995PMC228385

[18] Shopsin B, Gomez M, Montgomery SO, Smith DH, Waddington M, Dodge DE, Evaluation of protein A gene polymorphic region DNA sequencing for typing of *Staphylococcus aureus* strains. J Clin Microbiol. 1999;37:3556–63.1052355110.1128/jcm.37.11.3556-3563.1999PMC85690

[19] Mathema B, Mediavilla J, Kreiswirth BN. Sequence analysis of the variable number tandem repeat in *Staphylococcus aureus* protein A gene: *spa* typing. Methods Mol Biol. 2008;431:285–305.1828776410.1007/978-1-60327-032-8_22

[20] Chen L, Mediavilla JR, Oliveira DC, Willey BM, de Lencastre H, Kreiswirth BN. Multiplex real-time PCR for rapid staphylococcal cassette chromosome mec typing. J Clin Microbiol. 2009;47:3692–706. 10.1128/JCM.00766-0919726600PMC2772623

[21] Goering RV, Morrison D, Al-Doori Z, Edwards GF, Gemmell CG. Usefulness of *mec*-associated direct repeat unit (*dru*) typing in the epidemiological analysis of highly clonal methicillin-resistant *Staphylococcus aureus* in Scotland. Clin Microbiol Infect. 2008;14:964–9. 10.1111/j.1469-0691.2008.02073.x18828855

[22] Enright MC, Day NP, Davies CE, Peacock SJ, Spratt BG. Multilocus sequence typing for characterization of methicillin-resistant and methicillin-susceptible clones of *Staphylococcus* aureus. J Clin Microbiol. 2000;38:1008–15.1069898810.1128/jcm.38.3.1008-1015.2000PMC86325

[23] Saïd-Salim B, Mathema B, Braughton K, Davis S, Sinsimer D, Eisner W, Differential distribution and expression of Panton-Valentine leucocidin among community-acquired methicillin-resistant Staphylococcus *aureus* strains. J Clin Microbiol. 2005;43:3373–9. 10.1128/JCM.43.7.3373-3379.200516000462PMC1169154

[24] Johnson WM, Tyler SD, Ewan EP, Ashton FE, Pollard DR, Rozee KR. Detection of genes for enterotoxins, exfoliative toxins, and toxic shock syndrome toxin 1 in *Staphylococcus aureus* by the polymerase chain reaction. J Clin Microbiol. 1991;29:426–30.203765910.1128/jcm.29.3.426-430.1991PMC269793

[25] Diep BA, Stone GG, Basuino L, Graber CJ, Miller A, des Etages SA, The arginine catabolic mobile element and staphylococcal chromosomal cassette mec linkage: convergence of virulence and resistance in the USA300 clone of methicillin-resistant *Staphylococcus aureus.* J Infect Dis. 2008;197:1523–30. 10.1086/58790718700257

[26] Anthony RM, Connor AM, Power EG, French GL. Use of the polymerase chain reaction for rapid detection of high-level mupirocin resistance in staphylococci. Eur J Clin Microbiol Infect Dis. 1999;18:30–4. 10.1007/s10096005022210192711

[27] Harris SR, Feil EJ, Holden MT, Quail MA, Nickerson EK, Chantratita N, Evolution of MRSA during hospital transmission and intercontinental spread. Science. 2010;327:469–74. 10.1126/science.118239520093474PMC2821690

[28] Amaral MM, Coelho LR, Flores RP, Souza RR, Silva-Carvalho MC, Teixeira LA, The predominant variant of the Brazilian epidemic clonal complex of methicillin-resistant *Staphylococcus aureus* has an enhanced ability to produce biofilm and to adhere to and invade airway epithelial cells. J Infect Dis. 2005;192:801–10. 10.1086/43251516088829

[29] Edgeworth JD, Yadegarfar G, Pathak S, Batra R, Cockfield JD, Wyncoll D, An outbreak in an intensive care unit of a strain of methicillin-resistant *Staphylococcus aureus* sequence type 239 associated with an increased rate of vascular access device-related bacteremia. Clin Infect Dis. 2007;44:493–501. 10.1086/51103417243050

[30] Cho DT, Cha HY, Chang HH, Kim SW, Chung JM, Kim J, Risk factors for specific methicillin-resistant *Staphylococcus aureus* clones in a Korean hospital. J Antimicrob Chemother. 2006;57:1122–7. 10.1093/jac/dkl11416581917

[31] Li DZ, Chen YS, Yang JP, Zhang W, Hu CP, Li JS, Preliminary molecular epidemiology of the *Staphylococcus aureus* in lower respiratory tract infections: a multicenter study in China. Chin Med J (Engl). 2011;124:687–92.21518559

[32] Smyth DS, Wong A, Robinson DA. Cross-species spread of SCC*mec* IV subtypes in staphylococci. Infect Genet Evol. 2011;11:446–53. 10.1016/j.meegid.2010.12.00521172458PMC3046341

[33] Van Bellum A, Struelens M, de Visser A, Verbrugh H, Tibayrenc M. Role of genomic typing in taxonomy, evolutionary genetics, and microbial epidemiology. Clin Microbiol Rev. 2001;14:547–60. 10.1128/CMR.14.3.547-560.200111432813PMC88989

[34] Olive DM, Bean P. Principles and applications of methods for DNA-based typing of microbial organisms. J Clin Microbiol. 1999;37:1661–9.1032530410.1128/jcm.37.6.1661-1669.1999PMC84917

[35] Tumin R, Wang SH, Pancholi P, Khan Y, Hines L, Stevenson K. Relationship of social and medical factors to other antimicrobial resistance among methicillin-resistant *Staphylococcus aureus* strains. In: Abstracts of the 139th American Public Health Association Annual Meeting; Washington, DC, Oct 29–Nov 2, 2011. Washington (DC): American Public Health Association; 2011. Abstract 246417.

# Research article: Methicillin-Resistant *Staphylococcus aureus* Sequence Type 239-III, Ohio, USA, 2007–2009^1^

**DOI:** 10.3201/eid1810.120468

**Authors:** Shu-Hua Wang, Yosef Khan, Lisa Hines, José R. Mediavilla, Liangfen Zhang, Liang Chen, Armando Hoet, Tammy Bannerman, Preeti Pancholi, D. Ashley Robinson, Barry N. Kreiswirth, Kurt B. Stevenson, for the Prevention Epicenter Program of the Centers for Disease Control and Prevention

**Affiliations:** The Ohio State University Wexner Medical Center, Columbus, Ohio, USA (S.H. Wang, Y. Khan, L. Hines, A. Hoet, P. Pancholi, K.B. Stevenson);; University of Medicine and Dentistry of New Jersey, Newark, New Jersey, USA (J.R. Mediavilla, L. Chen, B.N. Kreiswirth);; University of Mississippi Medical Center, Jackson, Mississippi, USA (L. Zhang, D.A. Robinson);; and The Ohio Department of Health Laboratories, Reynoldsburg, Ohio, USA (T. Bannerman)

## Medscape CME Quiz

## Earning CME Credit

To obtain credit, you should first read the journal article. After reading the article, you should be able to answer the following, related, multiple-choice questions. To complete the questions (with a minimum 70% passing score) and earn continuing medical education (CME) credit, please go to www.medscape.org/journal/eid. Credit cannot be obtained for tests completed on paper, although you may use the worksheet below to keep a record of your answers. You must be a registered user on Medscape.org. If you are not registered on Medscape.org, please click on the New Users: Free Registration link on the left hand side of the website to register. Only one answer is correct for each question. Once you successfully answer all post-test questions you will be able to view and/or print your certificate. For questions regarding the content of this activity, contact the accredited provider, CME@medscape.net. For technical assistance, contact CME@webmd.net. American Medical Association’s Physician’s Recognition Award (AMA PRA) credits are accepted in the US as evidence of participation in CME activities. For further information on this award, please refer to http://www.ama-assn.org/ama/pub/category/2922.html. The AMA has determined that physicians not licensed in the US who participate in this CME activity are eligible for *AMA PRA Category 1 Credits*™. Through agreements that the AMA has made with agencies in some countries, AMA PRA credit may be acceptable as evidence of participation in CME activities. If you are not licensed in the US, please complete the questions online, print the certificate and present it to your national medical association for review.

## Article Title:  Methicillin-Resistant *Staphylococcus aureus* Sequence Type 239-III,  Ohio, USA, 2007–2009

## CME Questions

**1. You are seeing a 65-year-old woman admitted with newly-diagnosed methicillin-resistant *Staphylococcus aureus* (MRSA) bacteremia. Which of the following strains of MRSA are most common in the United States?**

A. USA 100 and MRSA ST239-III

B. USA 300 and MRSA ST239-III

C. USA 100 and USA 300

D. USA 600 and MRSA ST239-III

**2. As you evaluate this patient, what should you consider regarding clinical characteristics of MRSA ST239-III in the current study?**

A. Most cases of MRSA ST239-III were diagnosed in community hospitals

B. Ninety-five percent of cases of MRSA ST239-III were health care associated

C. There were no cases of MRSA ST239-III associated with implanted medical devices

D. Nearly all cases of MRSA ST239-III were bloodstream infections

**3. Which of the following statements regarding the treatment and prognosis of MRSA ST239-III in the current study is most accurate?**

A. There was broad susceptibility to nearly all antimicrobials tested

B. Rates of resistance to vancomycin and linezolid were approximately 90%

C. Rates of treatment failure of MRSA ST239-III were significantly higher compared with those associated with USA 100 and USA 300

D. Over 20% of cases of infection with MRSA ST239-III were fatal

**4. Which of the following statements regarding molecular typing of MRSA ST239-III infections in the current study is most accurate?**

A. There was a single repetitive element PCR (rep-PCR) pattern

B. There were 9 different pulsed-field gel electrophoresis (PFGE) patterns

C. Traditional PFGE testing could identify all of the bacteria subtypes

D. The cluster groupings from PFGE, rep-PCR, and dru methods were essentially identical

## Activity Evaluation

**Table:** 

**1. The activity supported the learning objectives.**				
Strongly Disagree				Strongly Agree
1	2	3	4	5
**2. The material was organized clearly for learning to occur.**				
Strongly Disagree				Strongly Agree
1	2	3	4	5
**3. The content learned from this activity will impact my practice.**				
Strongly Disagree				Strongly Agree
1	2	3	4	5
**4. The activity was presented objectively and free of commercial bias.**				
Strongly Disagree				Strongly Agree
1	2	3	4	5
